# An *in silico* approach in predicting the possible mechanism involving restoration of wild-type p53 functions by small molecular weight compounds in tumor cells expressing R273H mutant p53

**DOI:** 10.17179/excli2017-299

**Published:** 2017-12-08

**Authors:** Ibrahim Malami, Aliyu Muhammad, Imaobong C. Etti, Peter M. Waziri, Alhassan M. Alhassan

**Affiliations:** 1 Faculty of Pharmaceutical Sciences, Usmanu Danfodiyo University, Sokoto, Nigeria; 2 Department of Biochemistry, Faculty of Life Sciences, Ahmadu Bello University, Zaria, Nigeria; 3 Department of Pharmacology and Toxicology, Universiti of Uyo, Uyo, Nigeria; 4 Department of Biochemistry, Kaduna State University, Kaduna, Nigeria

**Keywords:** molecular docking simulation, R273H mutant p53, flavokawain B, alpinetin, curcumin, DNA-binding domain

## Abstract

R273H mutant p53 is a DNA-contact mutant that renders p53 dysfunctional due to a single substitution of Arg273 for His273. Rescuing R273 mutant p53 implies that a competent molecule would have to bind to the site of DNA-contact hot spots to complement the loss of contact with the DNA-binding domain. Here, curcumin, flavokawain B, and alpinetin were docked against the crystal structure of R273H mutant p53 *in silico*. Consequently, all the compounds bind to the cavity of R273H mutant p53 with a dissociation constant estimated to have 36.57, 70.77, and 75.11 µM for curcumin, flavokawain B, and alpinetin, respectively. Subsequently, each molecule was able to bind to the R273H mutant p53 by interacting with the DNA-contact hot spot Arg248 and mutant R273H, thereby compensating for the loss of direct contact with the DNA-binding domain. Furthermore, all the molecules were able to induce a direct contact with the consensus site of the DNA binding domain, thus maintaining DNA-contact residues with the DNA. The present findings offer preliminary indirect supporting evidence that small molecular weight compounds may certainly rescue DNA-contact mutant p53, which may lay a foundation for designing a competent and effective molecule capable of rescuing mutant p53 in tumor cells expressing R273H mutant p53.

## Introduction

TP53 is a tumor-suppressor protein that prevents cancer development through transcriptional regulation of target genes implicated in mutagenic cells potential for neoplastic transformation, cell cycle progression, DNA repair, and apoptosis (Bensaad et al., 2006[2]; Haupt et al., 2003[14]; Moll et al., 2006[28]). The loss of wild-type p53 activity by a mutation in the p53 gene leads to uncontrolled proliferation of cells (Parrales and Iwakuma, 2015[30]). Consequently, mutational inactivation of p53 is found in almost half of all human tumors and most are missense mutations occurring in the DNA-binding core domain of p53 (residues 92 - 292), resulting in the disruption of protein-DNA interaction (Tan et al., 2015[32]; Vijayakumaran et al., 2015[35]). The structure of p53 protein comprises of a complex domain structure reviewed in Freed-Pastor and Prives (2012[12]) and Joerger and Fersht (2007[17]), among which almost all oncogenic mutations found are within the DNA-binding core domain of p53 (Boeckler et al., 2008[3]). The six hot spot amino acid residues of the p53 (Arg175, Gly245, Arg248, Arg249, Arg273, and Arg282) located in or close to the DNA-binding surface are the most frequent mutations occurring in all human cancers. These hot spot sites are structurally classified either as contact (in which the amino acid residues Arg248 and Arg273 are directly in contact with the DNA, and any mutation will, therefore, lead to loss of contact with the DNA) or structural (in which the amino acid residues Arg175, Gly245, Arg249, and Arg282 play a pivotal role in the conservation of structural integrity to the surface of DNA-binding domain) (Joerger et al., 2005[19], 2006[18]).

Mutation in p53 gene ascribed to amino acid substitution not only lose the wild-type p53 functions but also acquire new gain-of-function (GOF) activities, thereby promoting tumorigenesis (Bykov and Wiman, 2014[9]; Liu et al., 2010[26]). Subsequently, high levels of mutant p53 protein found to accumulate in cancer cells, commonly due to the insusceptibility of dysfunctional mutant p53 to degradation by the murine double minute-2 (MDM2) (Bykov et al., 2005[7]). Therefore, mutant p53 have become a recent drug target for the development of novel therapeutics useful for cancer treatment. Moreover, restoring the wild-type p53 activity in cancer cells expressing mutant p53 will, therefore lead to a downstream transcription of target genes involve in cell cycle arrest and apoptosis. Prominently, different approaches have been exploited to restore the functional activity of p53 such as the reactivation of wild-type p53 activity via deletion of mutant p53 (Alexandrova et al., 2015[1]; Li et al., 2011[22]; Vakifahmetoglu-Norberg et al., 2013[34]; Wang et al., 2011[36]; Yan et al., 2014[39]; Yi et al., 2013[41]; Zhang et al., 2015[43]), rescuing mutant p53 via the second-site suppressor mutations (Joerger et al., 2005[19]), and the use of small molecular weight compounds (Bykov et al., 2005[8], 2002[6]; Issaeva et al., 2003[16]; Lambert et al., 2009[21]; Wischhusen et al., 2003[38]; Zache et al., 2008[42]). Contrary, the latter is quite challenging considering the loss of a DNA contact due to a single substitution of amino acid residue Arg273 for His273. However, to complement the loss of DNA-contact residues, a direct DNA contact is required with target molecule (Joerger and Fersht, 2007[17]).

R273H mutant p53 is one of the five most common mutations found in human cancers (Joerger et al., 2006[18]). Essentially, the arginine residue at position 273 which formed direct contact with the DNA response element is substituted with histidine, thus rendering p53 dysfunctional (Joerger and Fersht, 2007[17]). Hot spot mutation R273H has shown increasing resistance to anticancer agents such as cisplatin (Li et al., 2014[23]). In the present study, we have predicted for the first time the possible mechanism of restoring wild-type p53 functional activity involving R273H mutant p53. Whilst there have been previous studies on the view that small molecular weight compounds induced p53 transactivation of cancer cells harboring R273H mutant p53 (Lim et al., 2007[24]; Lin et al., 2015[25]; Malami et al., 2017[27]; Song et al., 2005[31]; Ye et al., 2015[40]) and restored their p53 functional activity (Bykov et al., 2005[8]; Lambert et al., 2009[21]; Weinmann et al., 2008[37]; Zache et al., 2008[42]; Demma et al., 2010[11]), the molecular mechanism involving restoration of wild-type p53 functional activity in tumor cells expressing R273H mutant p53 still remains unclear, and there has not been any attempt to understand the mechanism from the structural point of view. Recently, we have shown that the phytochemical compounds alpinetin and flavokawain B induced the activation and stabilization of p53 protein of HT-29 cell expressing R273H mutant p53 (Malami et al., 2017[27]), however, the molecular mechanism involved in triggering the wild-type p53 activity by the bioactive compounds is not clear. Therefore, the present findings highlight the probable use of natural compounds, curcumin, alpinetin and flavokawain, and their attributions in reactivating p53 function in tumor cells expressing R273H mutant p53. Concurrently, curcumin is additionally used in this study because of its restoration of p53 protein conformation in cancer cells harboring R175H and R273H mutant p53 (Garufi et al., 2013[13]).

## Materials and Methods

### Computer hardware and software

Protein-ligand docking simulation was run on a 4.00 GB RAM Intel® Core i5 2.5GHz with 64-bit Windows 10 Operating System (Acer Inc., New Taipei City, Taiwan). The 3D structure of bioactive compounds curcumin, flavokawain B and alpinetin compound used for the present study was drawn using ChemOffice version 5.1 (PerkinElmer, Waltham, MA, US) and saved as PDB file. The solution x-ray crystal structure of human R273H mutant p53 core domain protein (2bim.pdb, 1.98 Å resolution) (Joerger et al., 2006[18]) and solution x-ray crystal structure of DNA recognition in complex with wild-type p53 (2ahi.pdb, 1.85 Å resolution) (Kitayner et al., 2006[20]) were retrieved from the protein data bank (www.pdb.org) using Discovery Studio visualizer 4.5 (Accelryls, USA). Protein-ligand docking simulation was performed using Autodock software tools version 4.2 (Scripps Research Institute, US).

### Computational studies

#### (i) Protein-ligand docking 

Protein-ligand docking simulation study was initially performed using Autodock software tool. Water molecules and other heteroatoms were completely removed from the crystal protein, and all missing hydrogen atoms were added to the crystal protein. The ligand pdbqt file was prepared by adding gasteiger charges and removing non-polar hydrogen atoms to the ligands, whilst their charges were merged with their carbon atoms. The ligands roots were detected and defined their rotatable bonds, whilst their torsions were set to their respective numbers. Grid parameter file (gpf) was prepared by locating grid maps at 60 × 60 × 60 Å in xyz grid points, 0.375 Å spacing, and the position of the grid box was set centering in the active site residues surrounding R248A and R273H mutant p53 protein with xyz-coordinates of 98.591, 82.612 and 33.169 Å. A molecular docking simulation was conducted using energy evaluations of 2,500,000 for a number of 100 GA runs for each ligand with 150 population size. Lamarckian docking genetic algorithm was used to identify molecular binding sites and predict ligand binding energy, an inhibition constant as well as intermolecular energy (Morris et al., 2009[29]). A similar protocol was performed on each molecule used in this investigation.

#### (ii) Protein-nucleic acids docking

The second stage of the molecular docking studies involved protein-DNA docking performed on a novel NPDock web server accessed at http://genesilico.pl/NPDock (Tuszynska et al., 2015[33]). Solvent and other heteroatoms were completely removed from the crystal structure of the protein-DNA complex with the aid of DS visualizer. Their interaction was split and individually saved as PDB format. The result of the protein-ligand docking complex in the previous paragraph was prepared by manually relabeling all 'HETATM' to 'ATOM' in the PDB file using a Notepad++. This is to ensure all atoms in the protein and ligand contents in the PDB file are taken into consideration throughout the docking process as stipulated (Tuszynska et al., 2015[33]). Input PDB files are R273H mutant p53-ligand complex and crystal DNA structure. The number of decoys GRAMM generation was set at 20,000, whilst protein and DNA interface was at default settings. The simulation steps were set at 1,000 with a temperature at 15,000 K was used as the first step simulation, whilst 295 was used as the last step simulation temperature. The final top 10 clustering results were taken into consideration based on their scores, retrieved and analyzed with the aid of DS visualizer. The best interaction was chosen as the possible protein-DNA interaction.

## Results

### Assessing protein-ligand interaction

**Figure 1:**
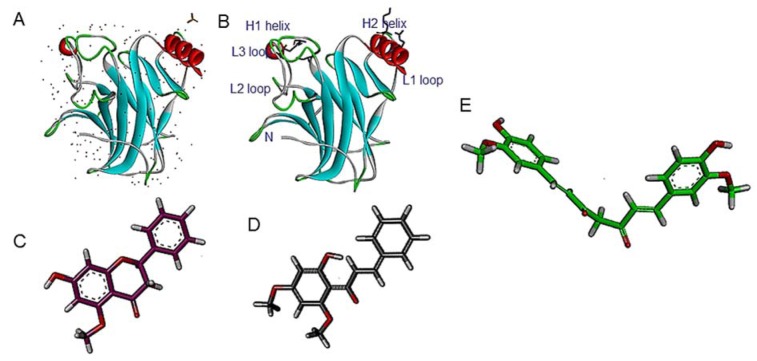
(A) X-ray crystal structure of solvated human R273H mutant p53 core domain (2bim.pdb) retrieved from protein data bank; (B) Cartoon diagram of the structure of R273H mutant p53 core domain; (C) 3D structure of alpinetin with purple carbon atoms; (D) 3D structure of flavokawain B with ash carbon atoms; (E) 3D structure of curcumin with green carbon atoms

Protein-ligand molecular docking was performed using free-energy and the AMBER force field against crystal structure of human R273H mutant p53 core domain (Figure 1(Fig. 1)). Subsequently, we are able to predict the binding of curcumin, alpinetin and flavokawain B to the target R273H mutant p53 protein. The bioactive compounds were successfully docked against R273H mutant p53 protein with an estimated dissociation constant of 36.57, 70.77 and 75.11 µM, respectively. Furthermore, their free binding energy was estimated at -6.05, -5.66 and -5.63 kcal/mol for curcumin, flavokawain B, and alpinetin, respectively. Final intermolecular energy and total internal energy were estimated at -9.04 and -1.62 kcal/mol for curcumin. On the other hand, alpinetin and flavokawain B were estimated to bind to p53 at -6.52 and -0.43, and -7.45 and -0.67 kcal/mol, respectively.

**Figure 2:**
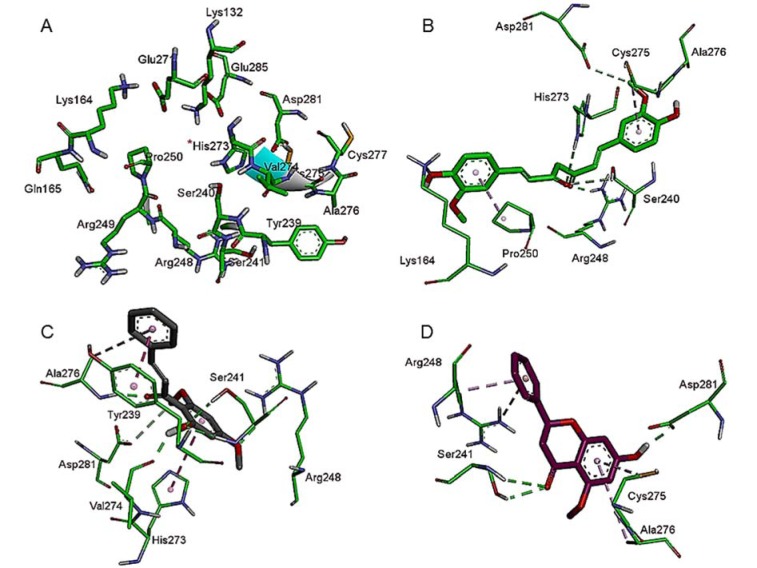
Intermolecular interactions formed in the R273H mutant p53 active site of DNA-binding core domain. (A) Structure of DNA-contact R273H mutant p53. Intermolecular interactions between R273Hmutant p53 and (B) curcumin (green carbon atoms); (C) flavokawain B (ash carbon atoms); (D) alpinetin (purple carbon atoms)

The present findings revealed a different binding mode of the compounds probably rescuing p53 functional activity. A molecular docking study involving curcumin results in the formation of eight intermolecular interactions with the active site residues in DNA-binding core domain of mutant p53 implicating six hydrogens and two π interactions. On the other hand, flavokawain B revealed a total of nine contacts forming six hydrogens and three π interactions. Both compounds form important interaction with DNA-contact R248A and mutant R273H (Figure 2B and C(Fig. 2)). Most prominently, the imidazolium group of a mutant side chain of His273 form hydrogen bonds with one of the carbonyl oxygen in the diketones group of curcumin, whilst the other carbonyl oxygen form a hydrogen bond with the guanidinium group of Arg248. Except for the mutant residue His273 which forms π interaction with its imidazolium group, flavokawain B also forms a hydrogen bond with the guanidinium group of Arg248 in a similar manner. Further docking analysis involving alpinetin reveals three hydrogens and four π interactions with the side chain residues in DNA-binding core domain of mutant p53. In contrast, alpinetin forms two π interaction with single DNA-contact hot spot Arg248 but not mutant His273 (Figure 2D(Fig. 2)).

**Figure 3:**
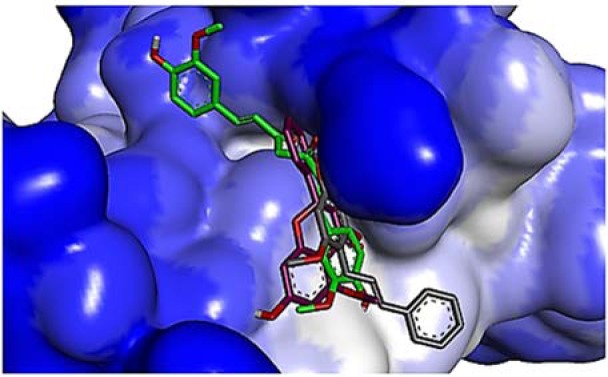
Different binding mode of ligand molecules to the molecular binding surface of DNA-contact R237H mutant p53. Curcumin is shown in green carbon atoms, flavokawain B in ash carbon atoms and alpinetin in purple carbon atoms.

The different binding mode of the bioactive compounds in the molecular binding surface of the R237H mutant p53 protein is shown in Figure 3(Fig. 3). The compounds were positioned surrounded by the amino acid side chains between the L3 loop and H2 helix of p53 protein harboring DNA-contact residues.

### Assessing protein-DNA interaction

**Figure 4:**
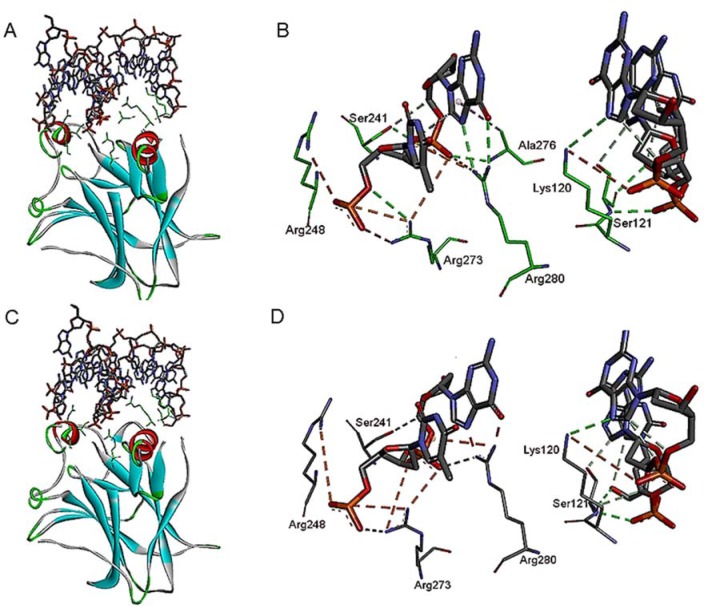
Cartoon structure of the wild-type p53 core domain in complex with DNA-binding domain. (A) Wild-type p53-DNA complex (2ahi.pdb) prior to molecular redocking simulation; (B) Wild-type p53-DNA complex following molecular redocking simulation. (C) Residues implicated in the direct DNA-contact of wild-type p53 to the DNA-binding domain prior to and following molecular redocking simulation, respectively.

GRAMM program and a combination of QUASI-DNP, DFIRE and Varani potential were employed for assessing protein-DNA molecular docking simulation. A redocking analysis of the split co-crystallized structure of a DNA complex with wild-type p53 was initially performed over the NPDock server to test the precision and efficiency of the protein-DNA docking simulation study used. Consequently, the side chains of DNA contact residue Arg248 and Arg273 forms a direct contact with the DNA phosphate backbone. Simultaneously, a direct contact is also formed from L3 loop via Ser241 side chain to the DNA phosphate backbone. Further to this, the residue Arg280 from H2 helix and the residues from L1 loop (Lys120 and Ser121) are bonded to the guanine bases (G8, G3, and G2) and DNA phosphate backbone (Figure 4(Fig. 4)). 

**Figure 5:**
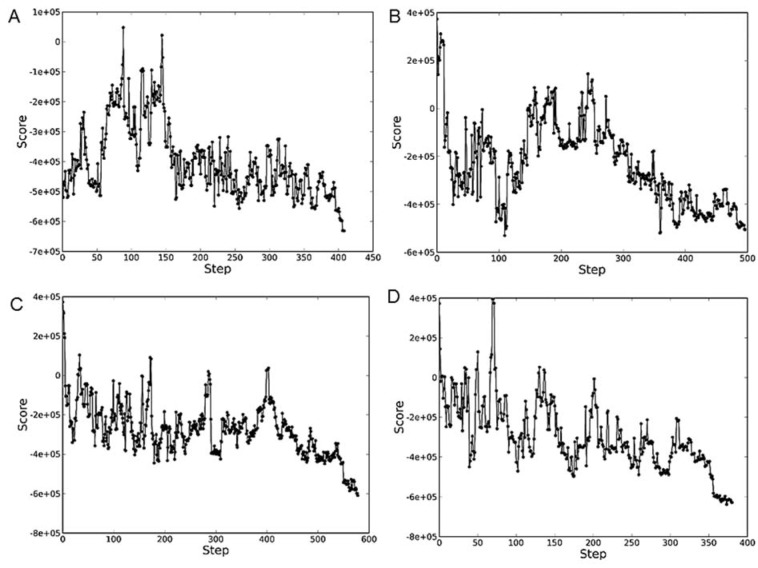
A plot showing variations in score during protein-DNA docking simulation involving (A) wild-type 53; (B) R273H mutant p53-curcumin complex; (C) R273H mutant p53-alpinetin complex; and (D) R273H mutant p53-flavokawain B complex.

The protein-DNA contact recognition retrieved from the redocking analysis is virtually identical to that retrieved from the original co-crystallized structure (2ahi.pdb). Therefore, the redocking analysis confirmed the validity and efficiency of running protein-DNA docking simulation study on the NPDock server. The plot scores variations verified during protein-DNA docking simulation is demonstrated in Figure 5(Fig. 5).

**Figure 6:**
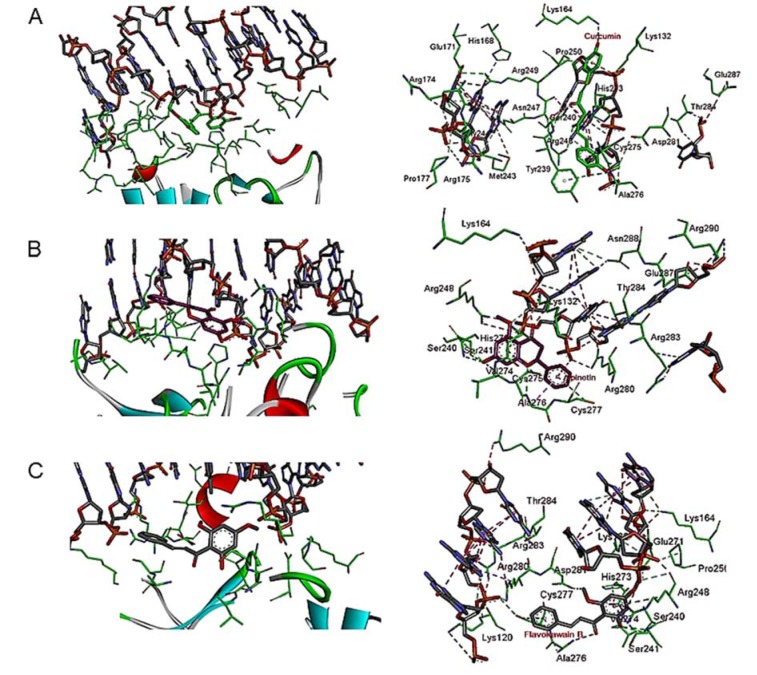
Intermolecular interactions of R273H mutant p53 core domain in complex with DNA-binding domain involving (A) curcumin as shown in green carbon atoms; (B) alpinetin as shown in purple carbon atoms; and (C) flavokawain B as shown in ash carbon atoms.

Subsequently, protein-DNA docking simulation involving curcumin revealed an interesting interaction between the side chains of p53 protein and the DNA-binding surface. Accordingly, curcumin is directly in contact with thymidine base (T7) and DNA phosphate backbone. Interestingly, a direct DNA contact is formed by a guanidinium group of Arg248 to the DNA bases and imidazolium group of His273 to the DNA phosphate backbone. Additional amino acid side chains that form direct contacts to the DNA bases include Glu171, Arg174, Arg175, Pro177, Gly244, and Arg249. The side chain of Lys132 is also bonded to the DNA phosphate backbone (Figure 6A(Fig. 6)). Similarly, alpinetin induced direct DNA contact with His273 and a guanidinium group of Arg248, thereby forming electrostatic interactions to the DNA phosphate backbone (Figure 6B(Fig. 6)). The ketone group of apinetin is bonded to the adenine base moiety (A6), hence maintaining a DNA contact between amino acid side chains of p53 and the DNA binding surface. Additionally, amino acid side chains that formed a direct contact with the DNA bases involves Thr284 and Asn288, whilst Lys132, Lys164, Glu287, and Arg290 are bonded to the DNA phosphate backbone. In contrast, flavokawain B forms a direct interaction to the DNA phosphate backbone, thereby inducing direct DNA-contacts with amino side chains in the DNA binding domain of p53 (Figure 6C(Fig. 6)). The side chain of DNA-contact residue R248A forms direct interaction to the DNA phosphate backbone (A6 and T7). Additionally, the side chain of Arg280 is bonded to the DNA bases, whilst nine residues Lys120, Lys164, Pro250, Glu271, Cys277, Arg280, and Arg283 are directly bonded to the DNA backbone.

**Figure 7:**
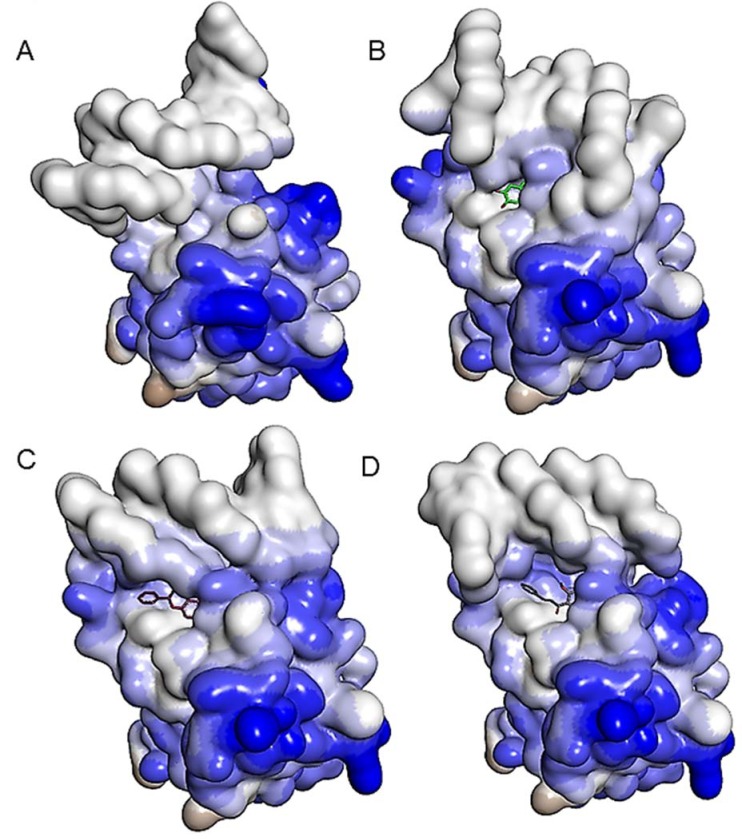
Binding mode of each of the ligand molecule in the molecular binding pocket of the R237H mutant p53-DNA complex. The binding mode of (A) wild-type p53-DNA complex; (B) R237H mutant p53-DNA complex involving curcumin; (C) R237H mutant p53-DNA complex involving alpinetin; (D) R237H mutant p53-DNA complex involving flavokawain B. A representation of atomic hydrophobicity showing a hydrophilic region in blue and hydrophobic region in brown.

Furthermore, substantial changes in the structure of a binding surface and DNA conformation was observed involving the bioactive molecules compared to the wild-type p53-DNA complex in Figure 7(Fig. 7). The binding mode of each of the bioactive compound observed inside the molecular binding pocket of R237H mutant p53-DNA complex structurally looks identical. However, the binding mode of mutant p53 to the DNA binding surface differ substantially among all the complexes. Additionally, slight changes in hydrophobicity are also observed in the structure of R273H mutant p53 involving all the compounds.

## Discussion

The present investigation attempt to predict the possible mechanism of reactivating wild-type p53 functional activity from the structural point of view. Whilst the induction of p53 transactivation of cancer cells harboring R273H mutant p53 by natural bioactive compounds curcumin, flavokawain and alpinetin have been demonstrated elsewhere (Lim et al., 2007[24]; Lin et al., 2015[25]; Malami et al., 2017[27]; Song et al., 2005[31]; Ye et al., 2015[40]), the molecular mechanism involving restoration of wild-type p53 functional activity is still not understood. In the present study, we employed the use of Autodock tools in protein-ligand docking using free-energy and the AMBER force field (Morris et al., 2009[29]). In the crystal structure of wild-type p53-DNA complex (2ahi.pdb), the guanidinium side chain of Arg273 forms a direct contact with the DNA phosphate backbone (Joerger et al., 2006[18]). In contrast, a mutation in DNA-contact residue Arg273 by His273, therefore, results in loss of interactions to the DNA-binding domain (Bullock et al., 2000[5]). In this study, molecular docking analysis involving the bioactive molecules have shown to interact with the residues in the DNA-contact binding pocket of mutant p53 protein.

The DNA-contact residue Arg273 plays an important role in mediating the p53 protein interaction with the DNA phosphate, the substitution of R273A by R273H will, therefore lead to an intense decrease in the binding affinity to the DNA (Kitayner et al., 2006[20]). Subsequently, the side chains of the DNA-contact residues Arg248 and Arg273 form a direct contact with the DNA phosphate backbone involving the bioactive molecules. In contrast to structural mutation, a mutation in p53 implicating R273H have been shown to have no effect on the thermodynamic stability of the protein core domain, and in the absent of the DNA-binding domain, the structure of the DNA-binding surface is still preserved (Bullock and Fersht, 2001[4]; Joerger et al., 2006[18]). Additionally, minor changes are usually induced in the nearby residues by a DNA-contact mutation R273H (Joerger et al., 2005[19]). Apart from the denatured state, any small molecule that is capable of stabilizing a protein would have to effectively bind to the folded state of the protein and thus, may restore the mutant functional activity (Joerger and Fersht, 2007[17]). In the present study, we have observed substantial conformational changes in the structure of DNA binding surface involving the bioactive compounds suggesting an entirely different binding mode to the DNA binding surface compared to that of the wild-type. The basis of DNA structural recognition by wild-type p53 implicates residues mainly from the L1 loop, H2 helix, L3 loop, and DNA phosphate from the minor and major groove of p53 DNA-binding core domain (Cho et al., 1994[10]; Kitayner et al., 2006[20]). In comparison, the present investigation was observed to have virtually near similar DNA recognition via a DNA-contact molecule bound to R273H mutant p53. Furthermore, all the bioactive molecules investigated possibly binds to a consensus site of the DNA binding domain, thus maintains DNA-contact residues to the DNA binding domain.

Findings from in-depth molecular docking simulation studies investigate the ability of curcumin, flavokawain B, and alpinetin to mediate direct reactivation of p53 functional activity essential in triggering cell cycle arrest and apoptosis. The present findings remain inconclusive as to whether the bioactive compounds could solely be responsible for rescuing mutant p53 via specific interaction with DNA binding core domain of R273H mutant p53. Obviously, the structurally modified compounds used in this study may have the potential to bind to the binding sites of the DNA binding core domain of mutant p53. There are several limitations observed in this study, for instance, the fact that these bioactive compounds could induce p53 protein expression in tumor cells expressing R273H mutant p53 as previously reported elsewhere (Lim et al., 2007[24]; Lin et al., 2015[25]; Malami et al., 2017[27]; Song et al., 2005[31]; Ye et al., 2015[40]) does not prove these molecules to have rescued R273H mutant p53. Secondly, their estimated Kd from the molecular docking simulation is poor, implying weak binding of the molecules to the mutant p53 and cannot have restored the mutant p53 binding affinity to the DNA binding surface. Nevertheless, the present findings offer a preliminary indirect supporting evidence that small molecular weight compounds may certainly rescue a DNA-contact mutant p53. This paves for designing a competent and effective molecule capable of restoring wild-type p53 functional activity of tumor cells expressing R273H mutant p53.
